# The Poisonous Effects of Amalgam Fillings

**Published:** 1885-08

**Authors:** Thomas Fletcher


ARTICLE VI.

### THE POISONOUS EFFECTS OF AMALGAM FILLINGS.

BY THOMAS FLETCHER, F.C.S.

The paper of Dr. Talbot on this subject reads like a paper written fifty years ago. If he had taken the trouble to test his own instructions for mixing he would have found that it is impossible to squeeze mercury out of the mixture. What he mistakes for mercury is a solution of some of the metals which should be left in the alloy, the removal of which almost invariably damages the amalgam. He would also have found that it is simply impossible to sqeeze the surplus mercury out of any compound amalgam, the quantity of mercury left in being quite double what is necessary to make a good amalgam. The "bond of union," if the mercury is in proper proportion and not in excess, is most certainly strong enough to prevent evaporation at ordinary temperatures. My own experiments with amalgams in the steam pipe of a high pressure steam engine showed conclusively that there was no loss of weight after three months' exposure; on the contrary, every plug showed a trifling increase in weight owing to surface oxidation.

Dr Talbot evidently puts all amalgams in the same basket, and believes that all amalgams shrink. If he had ever experimented with precipitated silver he would have been more cautious and more correct in his statements, and as to the question of porosity of plugs, it is quite possible to make either an amalgam or gold plug porous to any extent, as it is also possible to make either material watertight.

Dr. Austin's idea of lining a cavity with tin to take up surplus mercury is simply a makeshift and risky way of getting rid of what never ought to have been put in the amalgam, and the statement of Haswell quoted that "amalgams expand" is simply a proof that his experiments have been very limited and confined to one or two metals only.

The bulging of a filling which Dr. Talbot apparently takes as a proof of shrinkage, he will find has nothing whatever to do with this. If he will make a plug shaped as a true cube he will find it will slowly bulge on all six sides and the corners will draw inwards, showing a strong tendency to assume a globular form. That the amalgam is apparently hard proves nothing. Ice will flow under pressure and the tendency to assume a globular or spheroidal form after hardening varies with every different alloy.

That mercurial poisoning may occur in some cases where plugs are made with the grossest carelessness and an immense excess of uncombined mercury, may be possible, although I have never once, in twenty years' practice, seen such a case. But this, even if it does occur is a proof, not that amalgam *per se* is in fault, but that the dentist does not understand the material he is using. Any dentist who puts in a filling saturated with uncombined mercury had better discontinue using all amalgams until his education becomes more complete.—*Brit. Journal of Dental Science.*

